# Simvastatin and metformin inhibit cell growth in hepatitis C virus infected cells via mTOR increasing PTEN and autophagy

**DOI:** 10.1371/journal.pone.0191805

**Published:** 2018-01-31

**Authors:** José A. del Campo, Marta García-Valdecasas, Antonio Gil-Gómez, Ángela Rojas, Paloma Gallego, Javier Ampuero, Rocío Gallego-Durán, Helena Pastor, Lourdes Grande, Francisco J. Padillo, Jordi Muntané, Manuel Romero-Gómez

**Affiliations:** 1 Department of Digestive Diseases and CIBERehd, Valme University Hospital, Servicio Andaluz de Salud, Seville, Spain; 2 Department of Digestive Disease, Biomedicine Institute of Seville & CIBERehd, Rocío University Hospital, Seville, Spain; 3 Department of General Surgery, Rocío University Hospital, CSIC and University of Seville, Seville, Spain; University of Navarra School of Medicine and Center for Applied Medical Research (CIMA), SPAIN

## Abstract

Hepatitis C virus (HCV) infection has been related to increased risk of development of hepatocellular carcinoma (HCC) while metformin (M) and statins treatment seemed to protect against HCC development. In this work, we aim to identify the mechanisms by which metformin and simvastatin (S) could protect from liver cancer. Huh7.5 cells were infected with HCV particles and treated with M+S. Human primary hepatocytes were treated with M+S. Treatment with both drugs inhibited Huh7.5 cell growth and HCV infection. In non-infected cells S increased translational controlled tumor protein (TCTP) and phosphatase and tensin homolog (PTEN) proteins while M inhibited mammalian target of rapamycin (mTOR) and TCTP. Simvastatin and metformin co-administered down-regulated mTOR and TCTP, while PTEN was increased. In cells infected by HCV, mTOR, TCTP, p62 and light chain 3B II (LC3BII) were increased and PTEN was decreased. S+M treatment increased PTEN, p62 and LC3BII in Huh7.5 cells. In human primary hepatocytes, metformin treatment inhibited mTOR and PTEN, but up-regulated p62, LC3BII and Caspase 3. In conclusion, simvastatin and metformin inhibited cell growth and HCV infection *in vitro*. In human hepatocytes, metformin increased cell-death markers. These findings suggest that M+S treatment could be useful in therapeutic prevention of HCV-related hepatocellular carcinoma.

## Introduction

The natural history of hepatitis C infection frequently ranges from chronic infection to liver cirrhosis and liver cancer. The majority of HCC occurred on chronic liver diseases or cirrhosis [1]. The proportion of HCC caused by the HCV ranges from 50% to 70% in developed countries, depending on the prevalence of HCV-related cirrhosis [2]. In addition, HCV-related HCC was significantly related to poor survival [3]. Hepatitis C virus interacts with lipid metabolism affecting assembly and virion maturation [4,5]. Statins use resulted in 37% reduction in HCC risk in clinical practice [6]. Moreover, the use of statins increased survival rate in HCC-cirrhotic patients [7]. Statins mechanisms influencing liver cancer development and progression include anti-proliferative, pro-apoptotic, anti-angiogenic, immunomodulatory and anti-infective effects via: a) deregulation of cell cycle regulatory cyclins; b) increase in p19, p21 and p27 proteins; c) inhibition of Myc and AKT/mTOR pathways [6,8].

Metformin use in cirrhotics reduced HCC incidence [9]. This drug activates AMP-activated protein kinase (AMPK) and prevents mTOR phosphorylation [10]. The mTOR pathway is a major tumor-initiating pathway in hepatocellular carcinoma, with up-regulation seen in up to 50% of tumors [11]. Cultured cells treated with metformin showed cell cycle arrest at G0/G1 phase [12] and, in nude mice, this treatment caused xenograft HCC-tumor growth suppression [13].

Phosphatase and tensin homolog (PTEN) is a tumor suppressor which inhibits mTOR pathway. The most accepted hypothesis supports that HCV infection down-regulates PTEN to activate mTOR [10] and promotes HCC growth [14]. Translational controlled tumor protein (TCTP) has been classified as an oncogene and its effects include an anti-apoptotic role inhibiting BCL2-associated X Protein (Bax), down-regulates p53, induces a faster cell cycle progression which promotes higher mutation rate, and induces cytokines production that promote inflammation [15–17] suggesting TCTP could be a target for anti-cancer drugs.

The aim of the present study was to investigate the mechanisms by which metformin and simvastatin could prevent liver cancer in HCV infection using *in vitro* models based on Huh7.5 and human primary hepatocytes culture.

## Methods

### Cell culture and human primary hepatocytes

Huh7.5 cells (Apath LLC, New York, USA) were grown in DMEM culture medium supplemented with 10% fetal bovine serum (FBS), penicillin (100U/ml) streptomycin (100μg/ml) antibiotics, L-glutamine and non-essential aminoacids. Cells were incubated at 37°C, 5% CO_2_. Infective particles of JFH-1 were added to cell plate at 1 particle/cell, and simvastatin (2μM) (Sigma, San Louis, Missouri, USA) and/or metformin (2mM) (Acofarma, Barcelona, Spain) treatment were added 3 hours after cell seeding, and incubated together for 72 hours. To calculate cell viability, cells were seeded with three different concentrations of metformin (1mM, 2mM and 10mM) or simvastatin (1μM, 2μM and 4μM) over 24, 48 or 72 hours. Cell number and viability were determined using trypan blue test on a Neubauer chamber.

Human hepatocytes were prepared from liver biopsies obtained from 3 donors undergoing surgical resection of a liver tumor. Biopsy sampling was with informed consent of the patient, and the study was approved by the Rocio University Hospital’s Ethics Committee and was performed in accordance with approved guidelines. Hepatocytes isolation was based on the two-step collagenase procedure [18]. Cell viability was consistently 85%, as determined by trypan blue exclusion. Hepatocytes (8×10^6^ cells; 150,000 cells/cm^2^) were pooled and seeded at confluence on type I collagen-coated dishes (Iwaki, Gyouda, Japan) and maintained in a DMEM-Ham-F12: William’s E (1:1) supplemented medium for 12 h. The medium was then removed and replaced with a fresh culture medium supplemented, when indicated, with metformin (2mM) or simvastatin (2μM) for 72 hours.

### Cell-cycle arrest study

After treatment, Huh7.5 cells were trypsinized and 1×10^6^ cells were washed with PBS and fixed with 70% cold ethanol in PBS at -20°C overnight. After centrifugation (700 × *g*; 5 min), cells were resuspended in PBS containing 40 μg/mL PI and 100 μg/mL RNAse and incubated for 30 min at 37°C in the dark. Samples were then analyzed on a BD^™^CantoII flow cytometer (BD Biosciences) using BD FACS Diva6.0 software.

### Gene expression assays

Total RNA was extracted from cellular lysates using standard protocols. Reverse transcription reactions were performed using commercially available kits (Qiagen, Invitrogen, Carlsbad, CA, USA). Gene expression was analyzed by quantitative polymerase chain reaction (qPCR) using an Illumina Eco Real-Time PCR model cycler. *GAPDH*, *RRN18S*, *AKT*, *mTOR*, *PTP1B*, *PTEN*, *TCTP* and *MAPLC3B* primers were purchased from Qiagen (QuantiTect Primer Assays). The presence of JFH-1 RNA in cell cultures was determined by qPCR using specific primers (forward: CTGTGAGGAACTACTGTCT and reverse: CGCCCTATCAGGCAGTACCA) which targeted negative strand of HCV-RNA. JFH1 particle production in culture medium, was measured by COBAS^®^ Taqman^®^ HCV test v2.0.

### Protein analysis

Cells were disrupted using a M-PER Mammalian Protein Extraction Reagent kit (Thermo SCIENTIFIC) and total proteins were quantified using Bradford Assay. Total proteins (50ug) were used for western-blot analysis, and were loaded onto 10–12% SDS-PAGE (Mini-protean TGX Stain-free gels, BioRad, Hercules, California, USA) with prestained protein standards. Primary antibodies (mTOR, PTP1B, PTEN, TCTP, LC3B, p62, Caspase 3 and β-actin) were purchased from Cell Signaling Technology (Beverly, MA, USA) and anti-core antibody from Enzo Life Science (Postfach, Lausen, Switzerland). Proteins were detected by chemiluminescence, according to manufacturer’s instructions (WesternBright^™^ ECL, Advansta, Menlo Park, California, USA). Image analysis and quantification was performed using ChemiDoc^™^ MP Imaging System and ChemiDoc^™^ XRS+ software (BioRad).

### Statistical analysis

All experiments were performed in triplicate. Continuous variables were defined as means ± SD. Normal distribution was analyzed by Shapiro-Wilks test. Comparisons between groups were made using the Student *t*-test or ANOVA test for continuous and normal variables. Depending on variance homogeneity, Bonferroni or T2 Tamhane correction tests were used. Two-sided P values 0.05 were considered statistically significant (* p<0.05, ** p<0.01, ***p<0.001). Data were entered into a computerized database and analyzed using the SPSS package (SPSS 18.0 for Windows, Chicago, IL).

## Results

### Simvastatin and metformin decreased cell proliferation

**Fig 1:**
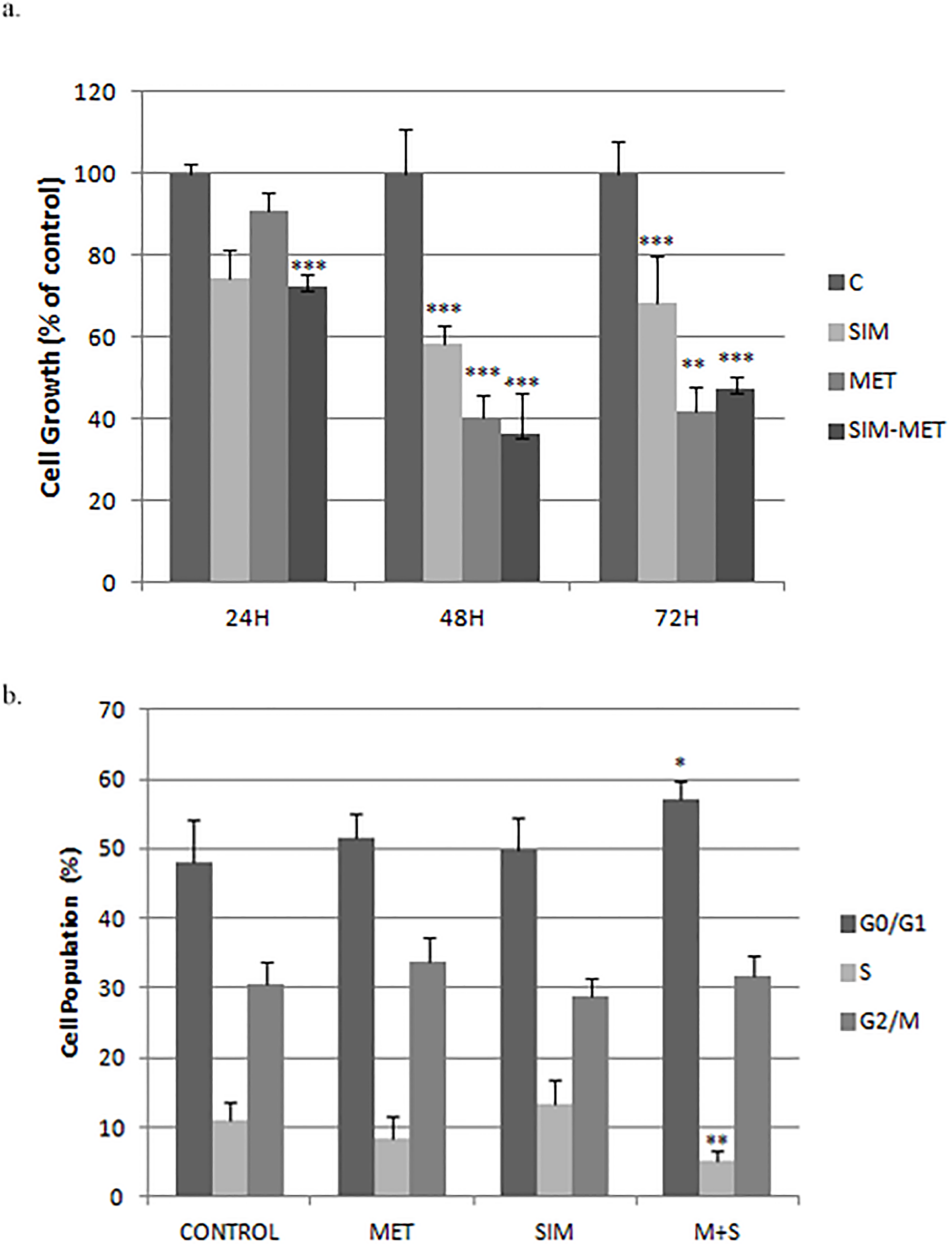
Cell viability of Huh7.5 cells treated with simvastatin and metformin. **A**: Percentage of cell kinetic in Huh7.5 treated with metformin (M), simvastatin (S) or combination (S: 2μM, M: 2mM). Cell number was quantified by Neubauer chamber. **B**: Percentage of Huh7.5 cell treated with these treatments in different phases of cell cycle measured by FACS analysis. * p<0.05, ** p<0.01, ***p<0.001.

Simvastatin and metformin impaired cell proliferation in a dose- and time-dependent manner in Huh7.5 cell (Fig 1a and S1 Fig). Simvastatin (4 μM) inhibited cell growth by 58%±3.23 (p = 0.009), metformin (10 mM) reduced cell growth by 84%±2.5 compared to vehicle treated cells after 72h treatment (p<0.001) (S1 Fig). Metformin (2mM) + simvastatin (2μM) combination did not increase inhibition rate compared to separated treatment (63%±3.24). For the remaining experiments, metformin 2 mM and simvastatin 2 μM were chosen.

To investigate the mechanisms responsible for cell growth inhibition FACS analysis was performed on propidium iodide stained cells to determine the effects of these drugs on cell cycle progression. As shown in Fig 1b, after simvastatin and metformin treatment for 72 hours the highest percentage of cells was found in the G0/G1 phase (+9.2% more cells in G0/G1 phase) while cells in S-phase decreased significantly (-4.56%), with no significant change in number of cells in G2/M phase (+1.36%).

### Metformin and simvastatin modified PTEN and TCTP expression

**Fig 2:**
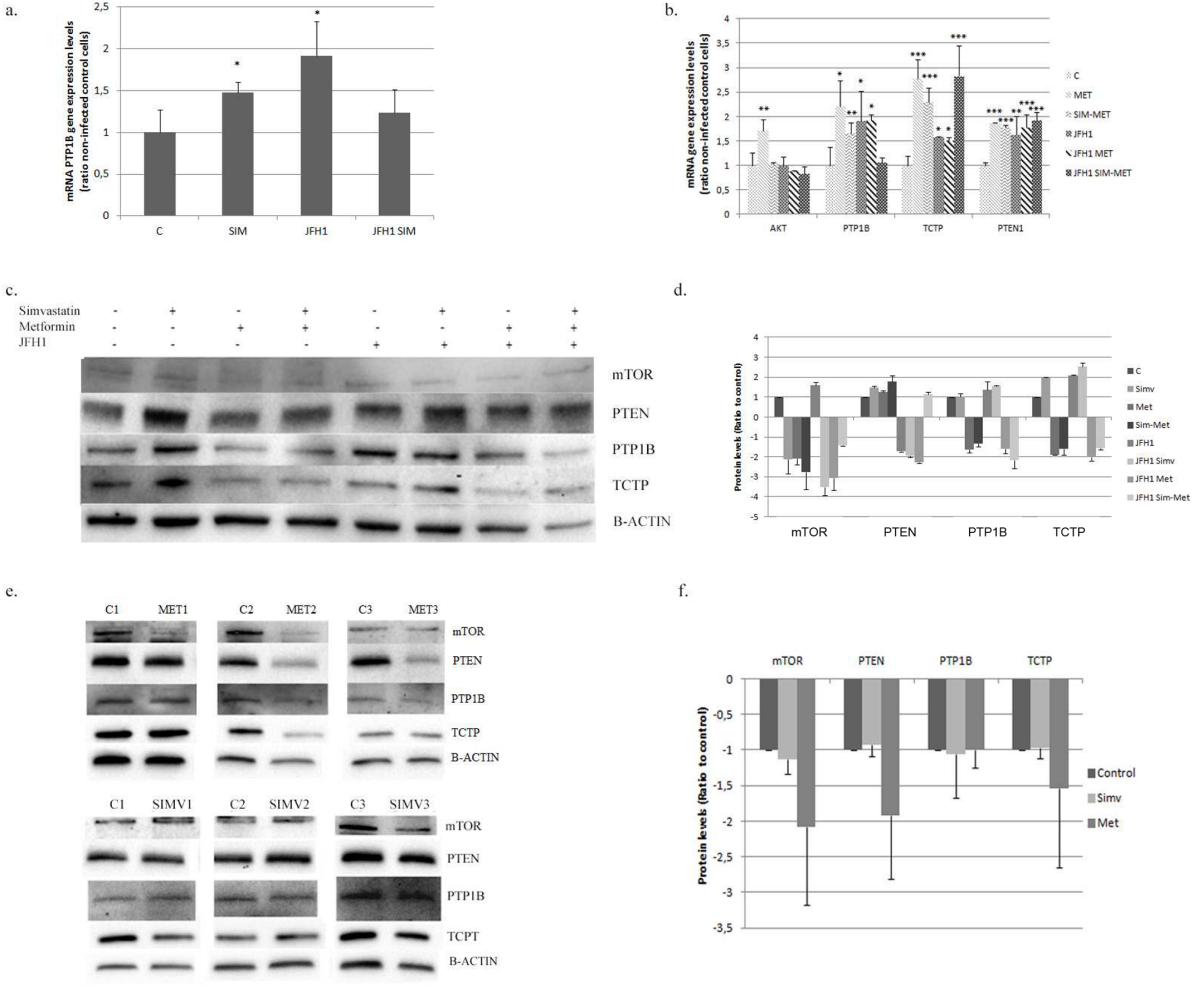
mTOR pathway modification in Huh7.5 cell and in primary hepatocytes. **A**: PTP1B gene expression in Huh7.5 cells infected with JFH1 particles (1 particle/cell) and treated 24 hours post-infection with simvastatin (S: 2μM) alone or in combination with metformin (SIM-MET; S:2μM, M: 2mM). **B**: Gene expression of mTOR pathway in Huh7.5 cells infected with JFH1 particles and treated at 24 hours post-infection with metformin (M: 2mM) alone or in combination with simvastatin. **C**: Protein expression by western-blot of mTOR pathway in control or JFH1 infected in Huh 7.5 cells treated with simvastatin (SIM; 2μM), metformin (MET; 2mM) or in combination (SIM-MET; S:2μM, M: 2mM) over 72 hours. All gels have been run under the same experimental conditions. **D**: Protein quantification of Huh7.5 using ChemiDoc^™^ MP Imaging System. **E**: Protein expression of the mTOR pathway in human primary hepatocytes treated with simvastatin (2μM) or metformin (2mM) quantified by western-blot of three different donors. **F**: Protein quantification of human primary hepatocytes using ChemiDoc^™^ MP Imaging System. * p<0.05, ** p<0.01, ***p<0.001.

In Huh7.5 cells treated with simvastatin (2μM) for 72h we detected increased tyrosine phosphatase1B (*PTP1B)* gene expression (1.5±0.2, p = 0.047) (Fig 2a). Metformin (2mM) treatment increased *AKT* (1.9±0.2), *PTEN1* (1.9±0.06), *PTP1B* (2.2±0.5) and *TCTP* (2.8±0.4) gene expression (Fig 2b). PTEN and TCTP protein expression were increased after simvastatin treatment (1.5±0.05 and 1.9±0.03-fold induction, respectively) while mTOR was inhibited 2.1±0.7 fold (Fig 2c and 2d). Metformin treatment down-regulated mTOR, PTP1B and TCTP protein expression (2.1±0.3; 1.64±0.2; and 1.9±0.02-fold inhibition, respectively) (Fig 2c and 2d).

Huh7.5 cells treated with simvastatin plus metformin showed induced gene expression of *PTP1B* (1.7±0.03 fold) *TCTP* (2.3±0.21 fold) and *PTEN1* (1.8±0.04 fold) (Fig 2b). The combination of both drugs decreased mTOR and TCTP protein expression (1.7±0.8; 1.6±0.2 fold inhibition, respectively) while PTEN was found increased (1.7±0.3) (Fig 2c and 2d)

Simvastatin had no significant effect on protein abundance in primary hepatocytes (Fig 2e and 2f). However, metformin down-regulated mTOR and PTEN protein levels (2.6±0.8 and 2.3±0.8-fold inhibition, respectively) (Fig 2e and 2f).

### HCV infection regulates PTEN, mTOR and TCTP

HCV infection increased gene expression of *PTP1B* (1.9±0.6), *PTEN1* (1.6±0.4) and *TCTP* (1.6±0.02) fold induction (Fig 2a and 2b). Moreover, TCTP and mTOR protein expression were also increased (2.1±0.3 and 1.6±0.13-fold induction, respectively), while PTEN was decreased (1.7±0.04-fold inhibition) (Fig 2c and 2d).

Huh7.5 infected cells treated with simvastatin (2μM) showed no significant changes in gene expression compared to control (non-infected). However, protein expression was significantly modified: TCTP was increased 2.8±0.2 fold while mTOR and PTP1B were down-regulated (3.5±0.4 and 1.6±0.05-fold inhibition, respectively) (Fig 2c and 2d).

Metformin down-regulated *TCTP* gene expression in infected cells (p = 0.006) (Fig 2b). Moreover, in infected cells, metformin also down-regulated TCTP, PTP1B, PTEN and mTOR protein expression.

In Huh7.5 infected cells, simvastatin + metformin treatment decreased *PTP1B* gene expression and increased *TCTP* (Fig 2b). Regarding protein expression, a significant inhibition of mTOR, TCTP and PTP1B was found (1.5±0.1; 1.6±0.04 and 2.1±0.4-fold inhibition), while PTEN1 was increased (1.2±0.1 fold), indicating a protective role (Fig 2c and 2d).

### Simvastatin and metformin inhibited HCV infection *in vitro*

**Fig 3:**
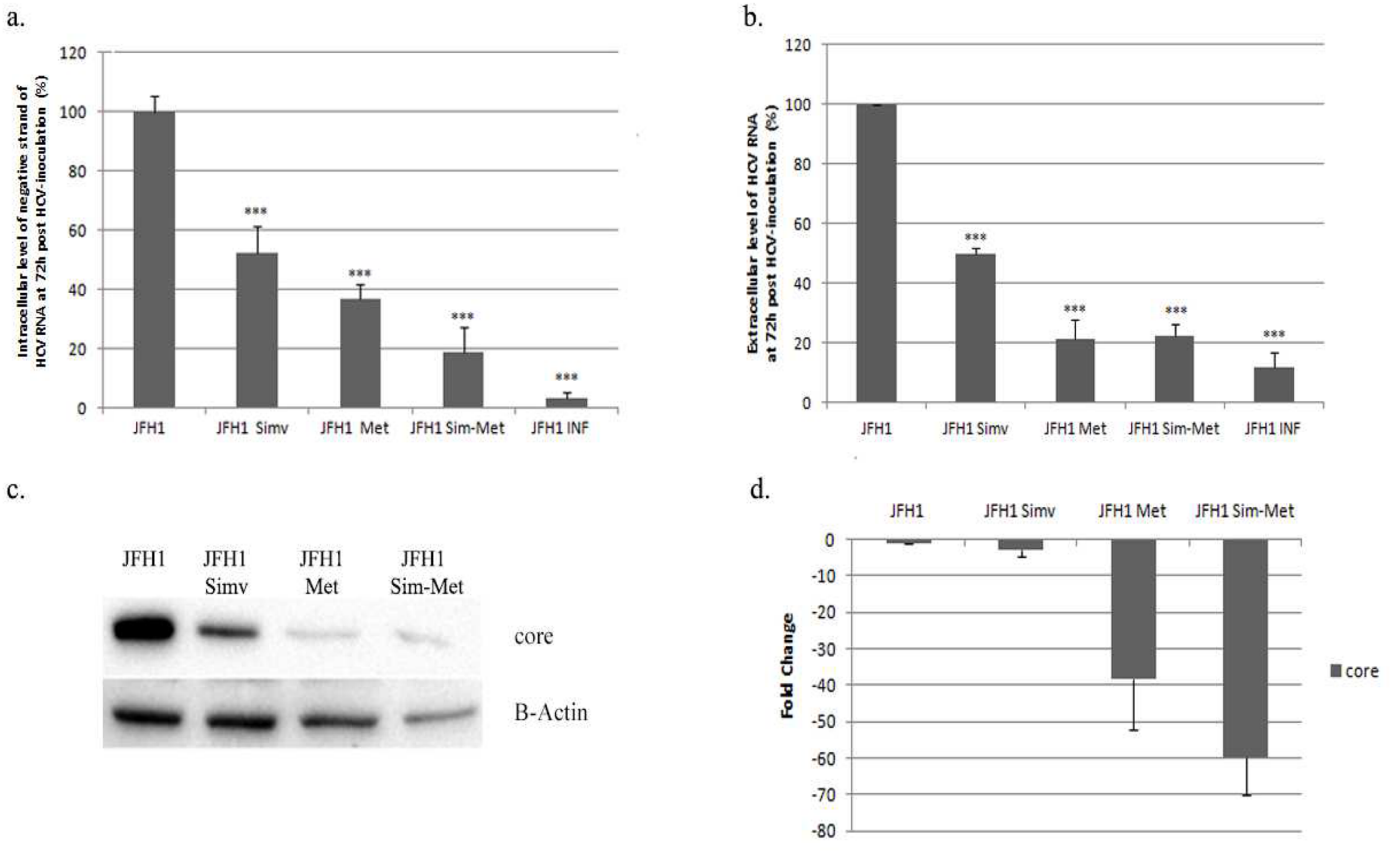
Metformin and simvastatin effects on HCV infection. **a**. Intracellular levels of negative strand of HCVRNA (%) in Huh7.5 cells infected with JFH-1and treated with simvastatin (SIM; 2μM), metformin (MET; 2mM) or in combination (SIM-MET; S: 2μM, M: 2mM), relative to complete infection (100% without any treatment). α-Interferon (500 IU/ml) was used as a positive control. **b**: Extracellular level of HCVRNA (%) measure in supernatant from Huh7.5 culture cells treated with simvastatin and metformin quantified by COBAS^®^ Taqman^®^ HCV test v2.0. **c**: core protein expression in Huh7.5 cells treated with simvastatin (2μM) or metformin (2mM) quantified by western-blot. **d**: core protein quantification using ChemiDoc^™^ MP Imaging System. * p<0.05, ** p<0.01, ***p<0.001.

HCV replication in Huh7.5 cells was hampered by metformin (2 mM), simvastatin (2 μM) and the combination of both drugs (Fig 3a). This effect was also found when measuring extracellular levels of HCV-RNA (Fig 3b). Metformin decreased core protein expression by 38.5±3.5 fold (Fig 3d). Simvastatin also down-regulated core protein expression (3.1±1.7-fold inhibition). Metformin treatment in HCV-RNA (up to 87.3%±8.8 inhibition rate) and core protein levels (60.0±10.0-fold inhibition) (Fig 3a, 3c and 3d). No significant changes were observed when comparing the effect of metformin alone or in combination with simvastatin in extracellular HCV-RNA down-regulation (Metformin: 21.4%±1.8, metformin+simvastatin: 22.4%±3.9) (Fig 2b).

### Metformin treatment activates LC3B and procaspase 3

**Fig 4:**
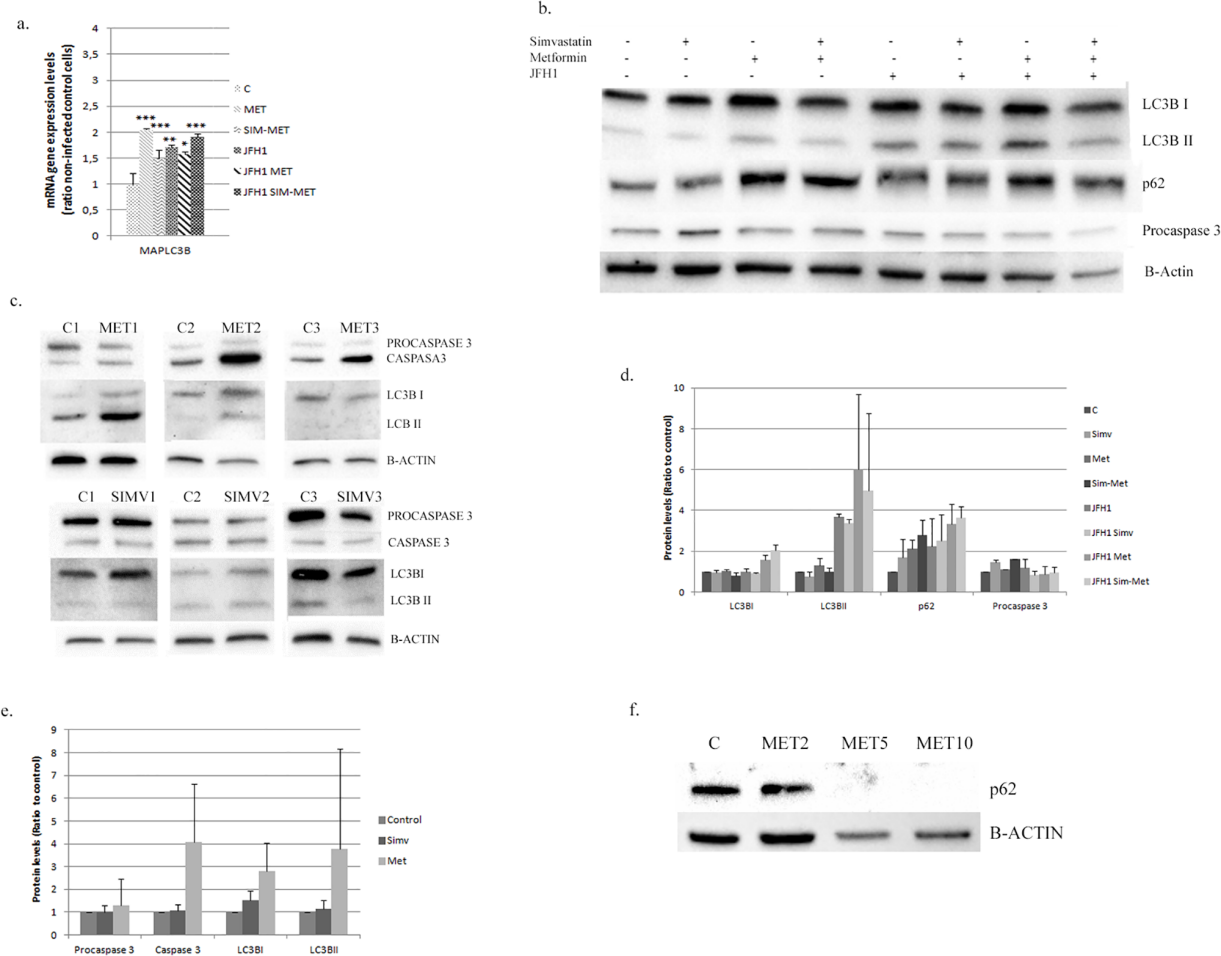
Autophagy modification in Huh7.5 cell and in primary hepatocytes. **A**: MAPLC3B gene expression in Huh7.5 cells infected with JFH1 particles (1 particle/cell) and treated 24 hours post-infection with metformin (M: 2mM) alone or in combination with simvastatin (SIM-MET; S:2μM, M: 2mM). **B**: Western-blot analysis of autophagy markers in control or JFH1 infected in Huh 7.5 cells treated with simvastatin (SIM; 2μM), metformin (MET; 2mM) or in combination (SIM-MET; S:2μM, M: 2mM) over 72 hours. All gels have been run under the same experimental conditions. **C**: Protein expression of autophagy markers in human primary hepatocytes treated with simvastatin (2μM) or metformin (2mM) quantified by western-blot of three different donors. **D**: Protein quantification of Huh7.5 using ChemiDoc^™^ MP Imaging System. **E**: Protein quantification of human primary hepatocytes using ChemiDoc^™^ MP Imaging System. **F**: p62 protein expressions in Huh7.5 cells treated with three different concentration of metformin (M2: 2mM, M5: 5mM and M10; 10mM). * p<0.05, ** p<0.01, ***p<0.001.

To analyze the effect of metformin on autophagy markers, we have evaluated changes in gene and protein expression in both, Huh7.5 cells and human primary hepatocytes. Light Chain 3B (LC3B) and Sequestosome 1 (p62) proteins are involved in autophagy. HCV infection induced *MAPLC3B* gene expression (1.7±0.1 fold) (Fig 4a) as well as protein expression of p62 and LC3BII (2.2±1.4 and 3.7±0.2-fold induction, respectively) (Fig 4b and 4d).

Huh7.5 cells treated with metformin (2mM) induced *MAPLC3B* gene expression (*MAPLC3B*: 2.0±0.03-fold) (Fig 4a). The expression of LC3BI and LC3BII were not modified, but p62 was found increased (2.1±0.4 fold) (Fig 4b and 4d). In infected cells (JFH-1) the effect of metformin treatment was more pronounced (p62: 3.3±0.9, LC3BI: 1.6±0.2 and LC3BII: +6.0±3.6-fold induction) (Fig 4b and 4d).

We tested whether metformin + simvastatin combination could have an effect in autophagy markers. Huh7.5 (mock and infected cells) were treated with these drugs and *MAPLC3B* gene expression was found up-regulated, regardless the presence of JFH1 particles (Fig 4a). LC3BI, LC3BII and p62 proteins were found up-regulated (3.7±0.5; 2.1±0.2; 4.9±3.8-fold change, respectively) (Fig 4b and 4d). We performed the same analysis in human primary hepatocytes, where simvastatin had no significant effect on these proteins (Fig 4a). However, metformin treatment increased p62 (+1.70±0.5); LC3BII (+5.6±4.2) and the activated protein caspase3 (4.1±2.5-fold induction) (Fig 4c and 4e), indicating autophagy events.

Metformin (5 and 10 mM) down-regulated p62 expression. This effect was not observed using lower concentration (2 mM) (Fig 4f).

## Discussion

In this work, we have shown that metformin and simvastatin in combination decreased hepatoma cell proliferation arresting cell cycle in G0/G1, inhibited TCTP and mTOR pathway, increased tumor suppressor PTEN and promoted autophagy in Huh7.5 cells infected by HCV or non-infected and in primary hepatocytes, suggesting that these drugs should be tested as a chemo-preventive option in HCC.

Our data demonstrated that cell growth was significantly decreased with simvastatin and metformin. Singh *et al*. have previously described an inhibition of cell proliferation with simvastatin treatment in Huh7 and HepG2 cells(8). Similar results have been obtained with metformin treatment alone [19]. In prostate cancer a synergic reduction of cell viability and an increase in G0/G1 phase was observed in metformin+simvastatin patients [20,21]. In our study, the combination of these drugs at 72h arrested cell cycle in G0/G1 phase, decreasing also cells in S phase. However, this combination failed to inhibit cell growth more than metformin alone at 48h.

A recent meta-analysis reported that the use of metformin reduced the ratio of liver cancer by 48%(22). A subgroup adjusted for the use of statins, showed a reduction in HCC incidence rate (OR = 0.75; 95% CI, 0.68–0.83; P<0.001), suggesting a synergistic effect of metformin and statins for liver cancer [22]. The increased cell number in G0/G1 phase and the cell reduction in S phase could be an explanation of these results.

MTOR plays an important role in HCC [11]. Metformin decreases mTOR expression through AMPK activation. Statins also inhibited mTOR in several tissues [23] as we observed in Huh7.5 cells, albeit this effect was not observed in primary hepatocytes. The role of statins on mTOR-Akt pathway was pointed out by Roudier et al.[24] through the inhibition of Akt phosporylation mediated by p53. Indeed, Huh7 cell lines harbor a mutated version of p53 protein [25].

Loss or down-regulation of PTEN expression leads to the activation of AKT/mTOR pathway promoting malignant progression [26]. Metformin down-regulates PTEN and enhances Protein kinase-β phosphorylation (AKT/PKB) [27]. However, we observed that simvastatin increases PTEN expression, alone or in combination with metformin. Up-regulation of PTEN with statins has been reported in breast cancer [28]. Others in vitro studies demonstrated that PTEN over-expression was able to reduce tumor proliferation in HepG2 [29], indicating a protective role for PTEN.

Protein-tyrosine phosphatase 1B (PTP1B) is an inhibitor that regulates phosphorylation levels of tyrosine kinase receptors [30]. The effect of PTP1B levels in tumor tissues has been controversial; in breast, colon and prostate cancer, tumor progression was promoted. In contrast, in esophageal cancer and lymphoma, tumor suppression has been reported [31]. In HCC, PTP1B seems to act as a tumor suppressor because it is down-regulated in tumors, while low PTP1B expression is associated with poor prognosis [30]. In our study, metformin inhibited PTP1B expression in Huh7.5 cells, but did not in primary hepatocytes or in combination with simvastatin.

TCTP is a pro-survival factor over-expressed in HCC. An increased TCTP is significantly associated with advanced HCC, and is an independent marker of poor prognosis [32]. TCTP plays an anti-apoptotic role, as an antagonist of pro-apoptotic Bax [17]. Lower TCTP levels reduce cell viability [33]. In this study, metformin (also in combination with simvastatin) inhibited TCTP in Huh7.5 cells and human primary hepatocytes (Fig 2c and 2e). These interesting data suggest that M + S should be considered for therapy in HCC.

HCV promotes HCC by several pathways. In our study, TCTP and mTOR were increased and PTEN was reduced. However, when infected cells were treated with M + S the opposite situation was found: TCTP/mTOR were down-regulated and PTEN was induced (Fig 2c and 2d). Peng et al. showed that mTOR pathway is upregulated by HCV NS5A to block apoptosis [34], and Peyrou et al. showed a PTEN inhibition by core protein genotype 3 [35]. Our data have shown that M+S treatment could reverse the scenario where HCV infection. We have previously reported that metformin inhibits viral replication [36]. Simvastatin also decreased viral infection as previously reported by Amemiya et al [37]. Metformin plus simvastatin combination yielded the highest level of viral infection inhibition (by 80% of viral replication) (Fig 3).

HCV infection induced autophagosome accumulation, but did not improve protein degradation in liver biopsies [38]. Autophagy regulates higher cholesterol level produced in HCV-infected cells, since hepatocytes have low cytoplasmic lipase levels. In genotype 3, it appears to be an inverse correlation between LC3BII/LC3BI ratio (an indicator of autophagy events), and the presence of microvesicular steatosis. This suggests that agents potentiating autophagy could prevent lipid accumulation in HCV patients [39]. Our results indicate an increased LC3BII in infected cells, but further induction of LC3B I and II was achieved with metformin alone or in combination with simvastatin. In an *in vitro* model of hepatocyte steatosis, a dysfunctional autophagy was induced and metformin treatment restored this process [40]. Metformin could serve as a pro-autophagic drug through activation of this process via mTOR inhibition [41] and increasing p53 [42]; an inhibitor and an activator of autophagy, respectively.

Autophagy is an adaptive pro-survival program under cellular starvation but, under continual cellular stress, autophagy could be a cell killing response process promoting autophagy cell death (also known as type II programmed cell death) [43]. The less aggressive HCC cell lines and tissues have much higher autophagy levels than the more aggressive HCC cell lines or tissues with recurrent disease [44]. Metformin treatment in primary hepatocytes induced caspase 3 as well as LC3BII–a primary marker for autophagy-. This effect has also been demonstrated in melanoma, colon and endometrial cancer [45]. In our study, p62 is found increased after metformin treatment (2 mM), indicating a blockade of the autophagy flux. However, Takahashi et al. observed that higher metformin concentration (5 and 10 mM) suppressed cancer cell growth via cell cycle arrest and complete autophagy [45]. We have found that higher metformin concentration (5 and 10 mM) decreased p62 protein levels (Fig 4f), indicating that previous autophagy flux blockade could be reversed by higher metformin concentration. Since no caspase activation was detected by metformin treatment, we can rule out that cell growth inhibition promoted by this drug in Huh7.5 cells can be due to cell cycle arrest in G0/G1 phase.

## Conclusions

In conclusion, our results showed that metformin + simvastatin combination inhibited HCV infection by reducing RNA negative strand and core protein. This treatment yields lower viral particles production, as well as cell viability inhibition by arresting cell cycle and down-regulating different oncogenic pathways. Metformin treatment inhibited mTOR pathway and the anti-apoptotic protein TCTP, while simvastatin increased the tumor suppressor PTEN. In addition, metformin treatment appears to enhance autophagy processes (probably in a dose-dependent manner) promoting cell growth inhibition. Taking into consideration all the results obtained, we propose that S+M therapy should be considered for patients with high risk of HCC development.

## Supporting information

S1 Fig Cell viability of Huh7.5 cells treated with simvastatin, metformin, or both. **A**: Huh7.5 cells were treated with different concentrations of simvastatin (S1: 1μM, S2: 2μM, S3: 4μM) for 72 hours, and cell number quantified by Neubauer chamber; **B**: Cell viability kinetic in cells treated with metformin (M1: 0.5mM, M2: 2mM, M3:10mM).
(TIF)
