# Supplementary figures and images for: Simvastatin and metformin inhibit cell growth in hepatitis C virus infected cells via mTOR increasing PTEN and autophagy
Source: PLoS One. 2018 Jan 31;13(1):e0191805. doi: 10.1371/journal.pone.0191805 (PMC5791999; doi:10.1371/journal.pone.0191805)

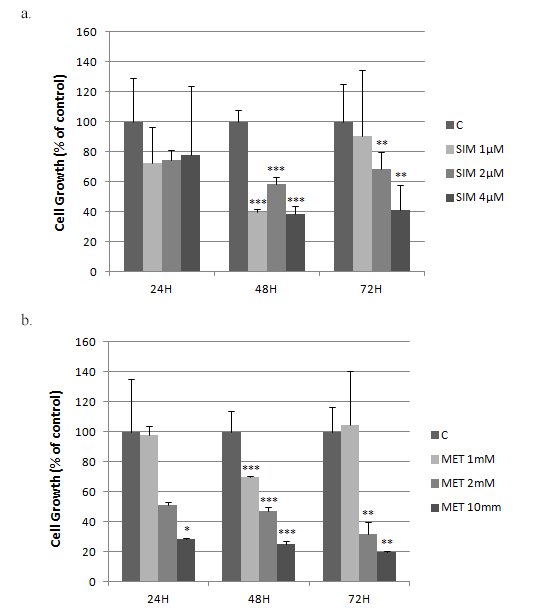

Supplement: S1 Fig — A: Huh7.5 cells were treated with different concentrations of simvastatin (S1: 1μM, S2: 2μM, S3: 4μM) for 72 hours, and cell number quantified by Neubauer chamber; B: Cell viability kinetic in cells treated with metformin (M1: 0.5mM, M2: 2mM, M3:10mM). (TIF) [file pone.0191805.s001.tif]
